# An Anti-UAV Long-Term Tracking Method with Hybrid Attention Mechanism and Hierarchical Discriminator

**DOI:** 10.3390/s22103701

**Published:** 2022-05-12

**Authors:** Feng Cheng, Zhibo Liang, Gaoliang Peng, Shaohui Liu, Sijue Li, Mengyu Ji

**Affiliations:** 1 State Key Laboratory of Robotics and System, Harbin Institute of Technology, Harbin 150000, China; 19b908022@stu.hit.edu.cn (F.C.); pgl7782@hit.edu.cn (G.P.); lisijue@hit.edu.cn (S.L.); 19b308008@stu.hit.edu.cn (M.J.); 2 Department of Computer Science and Technology, Harbin Institute of Technology, Harbin 150000, China; zbliang@hit.edu.cn

**Keywords:** anti-UAV, long-term tracking, attention mechanism, discriminator, Siamese network

## Abstract

To prevent unmanned aerial vehicles (UAVs) from threatening public security, anti-UAV object tracking has become a critical issue in industrial and military applications. However, tracking UAV objects stably is still a challenging issue because the scenarios are complicated and the targets are generally small. In this article, a novel long-term tracking architecture composed of a Siamese network and re-detection (SiamAD) is proposed to efficiently locate UAV targets in diverse surroundings. Specifically, a new hybrid attention mechanism module is exploited to conduct more discriminative feature representation and is incorporated into a Siamese network. At the same time, the attention-based Siamese network fuses multilevel features for accurately tracking the target. We further introduce a hierarchical discriminator for checking the reliability of targeting, and a discriminator-based redetection network is utilized for correcting tracking failures. To effectively catch up with the appearance changes of UAVs, a template updating strategy is developed in long-term tracking tasks. Our model surpasses many state-of-the-art models on the anti-UAV benchmark. In particular, the proposed method can achieve 13.7% and 16.5% improvements in success rate and precision rate, respectively, compared with the strong baseline SiamRPN++.

## 1. Introduction

Due to the significant advantages of high efficiency and low cost, unmanned aerial vehicles (UAV) have gained emerging interest in various applications such as military, transportation, logistics, and security [1,2]. Nevertheless, the illegal and unregulated use of UAVs also raises many potential hazards such as disturbing civil aviation and invading privacy, which may cause great threats to public safety. Moreover, it is difficult to effectively and rigorously supervise UAVs [3]. Such safety threats make anti-UAV systems necessary.

Target tracking is the premise of building an anti-UAV system. Using computer vision algorithms is a realistic solution to monitor UAVs, as video image signals are sensitive, accurate, and robust to interference. Specifically, visual tracking is one of the fundamental tasks in computer vision [4]. Given the initial template of an object in the first frame, the goal of tracking is to accurately identify and locate the object in sequential frames. In the case of UAV visual target tracking, video scenarios are complicated and variable, and the objects to be tracked are small and not obvious in appearance. This causes the tracker to suffer from numerous challenges, such as deformation, occlusion, targets moving out of view, and fast motion. Therefore, accurately tracking UAV objects is a challenging issue that must be addressed.

According to the characteristics of UAV video, anti-UAV tracking can be regarded as a long-term tracking task. The most important characteristic in the long-term is that the target in the tracking sequence can be completely occluded or out of the field of view. In recent years, many attempts have been addressed to improve the performance in UAV scenarios. Conventional tracking methods based on correlation filtering [5] (CF) or deep learning [6] (DL) have been widely used. However, some shortages, such as the deficiency of model learning ability or the constraint of real time, limit the application of these methods.

Recently, the Siamese-network-based trackers have made significant development, which treats object tracking as a matching problem. The core idea of a Siamese network is to learn a general similarity mapping between the target template and the search region. In this way, the tracking problem can be transformed into a similarity calculation of the search area and target. Siamese trackers usually adopt fully convolutional networks trained end to end, which have achieved outstanding performance in accuracy and real time. SiamFC [7], a pioneering work, was proposed in 2016 and reached a high frame rate of 58 fps. CFNet [8] integrates correlation filtering (CF)and a Siamese network, where the correlation filtering is constructed as a subnetwork layer. SiamRPN [9] introduced the region proposal network (RPN) [10] based on SiamFC [7]. However, Siamese-based models are trained completely offline and the templates cannot be updated online [7,9,11,12], which makes it difficult for them to catch up with appearance changes. In addition, although researchers have proposed many approaches for template establishment and updating, these methods generate feature maps only based on the target. The contextual information between positive and negative samples is entirely discarded, while some background features are beneficial to distinguish the target from similar disturbances. Even now, it is still challenging to achieve high-quality performances in practical tracking scenarios.

Long-term tracking is a more complex task. Tracking results may degenerate into unreliability when the object is heavily obscured or even out of view. Therefore, tracking-by-detection and redetection methods become essential in this case. There are two critical issues when designing a redetection method: how to evaluate the reliability of the tracking results and when to choose a better result to replace the original one. Many long-term trackers combine correlation filtering with image detection methods [13,14,15] and directly construct the evaluation criterion for tracking reliability based on the response maps generated by CF. Previously, [16,17] proposed a detection module using a global search strategy based on a Siamese network. The above long-term tracking methods are more robust in practical applications. However, detection at each frame is quite a costly operation. Accurately identifying if a tracker has failed is a vital problem; this determines whether the detection algorithm can be activated correctly. At present, many methods [13,14,15] designed reliability estimation only based on correlation filters with hand-crafted features and failed to take full advantage of deep learning in feature representation capabilities.

Motivated by the above analysis, an anti-UAV long-term object tracking method composed of a Siamese network and a redetection module which is configured with a hybrid attention mechanism and hierarchical discriminator is proposed. Specifically, the hybrid attention module is trained to enhance the feature learning capability of the Siamese network to generate more discriminative object representations. In addition, to solve the issue of re-locating the UAV target and template updating in long-term tracking, we exploit a hierarchical discriminator to generate response maps for target localization based on the output of the Siamese network and put in place a reliability criterion to evaluate the credibility of the response maps. When the output results indicate low confidence, the algorithm activates the re-detection module and updates the template.

The main contributions of this work are summarized as below:

(1) A multiple hybrid attention mechanism (MHAM) is introduced to compute channel–spatial attention and self-attention jointly, which can not only enhance interdependent channel-wise features and spatial features, but also capture rich contextual interdependency between the target and the background in UAV tracking tasks.

(2) A hierarchical discriminator (HDC) is adopted to estimate the credibility of tracking and redetection results and act as a trigger for detector and template updating. We apply the detector and updater to construct a modular framework for handling localization and drift tracking failures.

(3) The proposed method outperforms state-of-the-art methods on the anti-UAV benchmark. Our framework achieves the improvements of 13.7% in distance precision and 16.5% in overlap precision compared with SiamRPN++ [18] and realizes performance improvement (beyond 5%) against other SOTA trackers.

## 2. Related Work

In this section, we briefly discuss some classical and relevant methods, including correlation tracking, Siamese-based tracking and long-term tracking.

### 2.1. Correlation Tracking

In recent years, object tracking has gradually become a hot topic in the field of computer vision, and with the development of target tracking technology, correlation filtering and Siamese networks have received extensive attention. Correlation-filter-based methods dramatically reduce computation complexity by transforming the complicated computation under the time domain to the frequency domain. Bolme et al. [19] introduced the correlation filter into object tracking algorithms and first proposed MOSSE. Joo et al. [20] proposed CSK to replace dense sampling with circular sampling and improved the speed of ridge regression operation by diagonalizing the Fourier transformation of the cyclic shift matrix. Henriques et al. [5] exploited KCF by extending the single-channel CSK tracker to multiple channels. Danelljan et al. [21] and Yang et al. [22] applied target-scale adaptive estimation methods based on scale filters to improve the robustness of the model.

However, traditional correlation-filter-based methods mainly adopt hand-crafted features, which limits their performance to broader applications. In view of deep neural networks’ efficient feature representation capabilities, increasing attention has been attracted to fusing deep learning and correlation filters [23,24,25]. However, they still suffer from low accuracy or low speed under complex scenes.

### 2.2. Siamese-Based Tracking

In the past few years, many works have begun to focus on Siamese-based trackers due to their exceptional precision and speed. Bertinetto et al. [7] proposed SimFC, which adopted cross-correlation to a fully convolutional Siamese network, and the network was trained end-to-end and was capable of inferences in real time. Valmadre et al. [8] introduced correlation filter layers into the template branch and generated a robust feature map against translations. Inspired by Faster R-CNN [10], Li et al. [9] presented SiamRPN by applying the RPN module to object tracking tasks. SiamRPN [9] considered single-target tracking as a one-shot detection. It avoided the repeated computation of multiscale feature maps and significantly improved scale estimation accuracy compared with SiamFC [7]. Zhu et al. [26] proposed DaSiamRPN based on SiamRPN [9], which ameliorates the imbalance between positive and negative samples through data augmentation. The experiment results showed obvious improvements in long-term tracking. To break through the limitations of the shallow backbone network, SiamDW [12] and SiamRPN++ [18] adopted a deeper feature extraction network (ResNet50). Encouraged by the success of SiamRPN++ [18], many advanced models have been proposed. SiamBAN [27] exploited an anchor-free Siamese network. SiamMask [28] introduced image segmentation into the Siamese-based tracker. However, for the siamese model, tracking template updating is decisive to refrain from being disturbed by interferences. Recent models have developed various template updating strategies to solve the problem caused by changes in the appearance of targets [29,30,31]. The common solution is to employ historical tracking results for weighted fusion [32,33]. However, for Siamese trackers, high-quality performance is still unavailable in challenging real-world scenarios.

### 2.3. Long-Term Tracking

Long-term tracking refers to the task of continuously locating an arbitrary target in a relatively long video sequence. As the target may temporarily disappear, the critical problem is retrieving the target after a period of absence or tracking failures. Therefore, long-term tracking has more significant potential in real-world applications. TLD [34] decomposed the task into three stages: tracking, learning, and detecting. While tracking failed, the detection module was activated to re-locate the lost target. MUSTer [35] was composed of short-term and long-term memory subnets. Specifically, an integrated correlation filter was exploited as a short-term tracker, and the long-term store was employed to control short-term memory. However, MUSTer [35] lacked an evaluation mechanism for the reliability of tracking states, and redetection was triggered at each frame. LCT [36] trained a DCF-based tracker to estimate variations in the target’s location and scale and proposed an online random ferns detector to redetect lost targets. Wang et al. [14] and Tang et al. [15] exploited a DCF-based long-term tracking method which consisted of tracking-by-detection and redetection modules.

With the rapid development of deep learning, many DL-based long-term trackers are proposed. SPLT [37] and MBMD [16] adopted a Siamese-based tracker as a regression network. LTMU [38] proposed a short-term tracker combining SiamMASK [28] and MDNet [7] and fed the sequential information of the tracking results into long-short-term memory (LSTM) to decide whether to redetect or update. Motivated by the SiamRPN [9] framework, GlobalTrack [17] adopted a global search strategy for long-term tracking. However, the model updating strategies and redetection methods of long-term anti-UAV trackers are still relatively simple. Meanwhile, few works focus on fixing attention distribution to obtain more powerful semantic features. In this work, we present a hybrid-attention mechanism to conduct more discriminative feature representation. The hierarchical discriminator is proposed based on SiamRPN [9] for redetection and template updating in long-term tracking tasks.

## 3. Proposed Algorithm

### 3.1. Architecture

**Figure 1:**
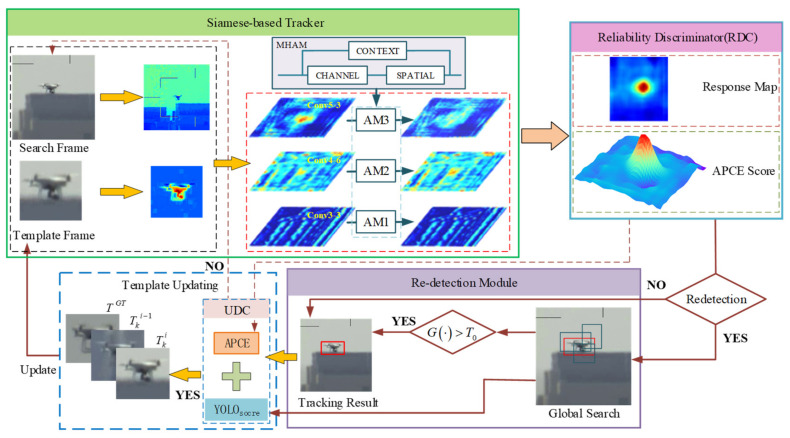
An overview of the proposed tracking algorithm. It consists of a Siamese-based tracker, hierarchical discriminator, redetection module, and template updating module. The features extracted from the backbone are fed to the attention module to generate the response map and estimate the object’s position. If the results are unreliable, the redetection module will be activated.

In this work, we propose an anti-UAV long-term tracking approach based on two components: (1) multihybrid attention module consisting of spatial, channel, and context attention, which is inserted into a Siamese network and learned offline. (2) hierarchical discriminator consisting of reliability discriminator (RDC) and updating discriminator (UDC). The above components are integrated into a multi-mission framework, which is illustrated in Figure 1.

The proposed framework can be divided into four parts: Siamese-based tracker, hierarchical discriminator, re-detection module, and template updating. Correspondingly, the UAV tracking process can be divided into four stages: tracking, evaluation, detection, and updating. During the tracking phase, the Siamese network extracts the features of the target template and search region and generates the response maps of tracking results through feature similarity matching. The Siamese-based tracker adopts a multiscale fusion and attention mechanism in the feature extraction network. Specifically, our proposed MHAM is utilized to obtain a more discriminative object representation which consists of three hybrid attention modules. The algorithm obtains an initial tracking result of the UAV and then proceeds to the evaluation phase. The hierarchical discriminator takes the response map from the tracker as input, then outputs the score for the tracking state in the current frame. The current tracking result will be output directly if the tracking state is considered reliable. Otherwise, the redetection module will be activated. During the detection phase, a redetection network is used to perform a global search of the current frame image and generates a detection result. If the result is reliable, the original tracking result will be replaced and the new target will be used as a sample for template update. During the updating phase, an updating discriminator is designed to assess the accuracy of tracking or detection results and decide whether the target template needs to be updated. Highly reliable tracking and detection results will be stored as samples in a memory set. Subsequently, the updating module retrieves information related to the object from the memory set. The synthetic feature map is generated to replace the current template.

### 3.2. UVA Target Tracking Based on Siamese Tracker

**Figure 2:**
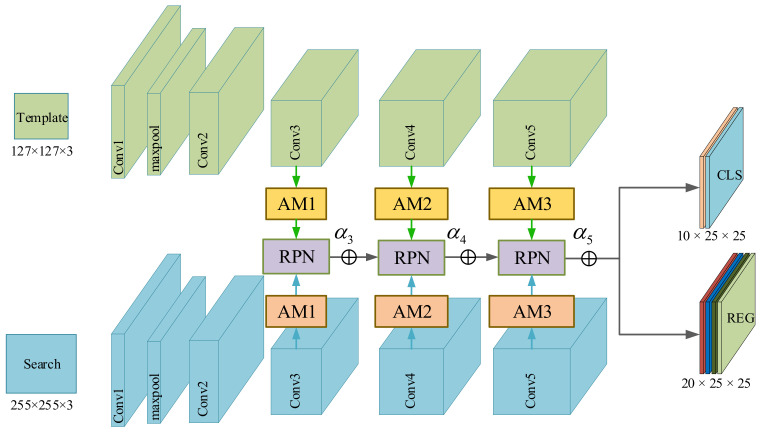
An overview structure of the Siamese network of RPN. It adopts ResNet50 as the backbone, and multilayer features are utilized for the layers conv3-3, conv4-6, and conv5-3. We add the attention modules to SiamRPN++ [18] between the backbone and RPN (adapted with permission from Ref. [18]. 2022, Li, B, et al).

**Figure 3:**
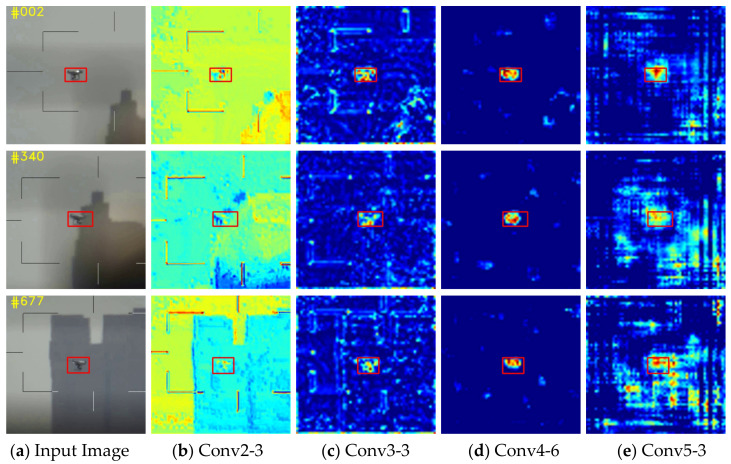
Visualization of convolutional layers of ResNet50. The first column is three frames from one UAV video. The second to fifth features are output from convolutional layers conv2-3, conv3-3, conv4-6, and conv5-3. Note that when the appearance of the target changes, the shallow feature fluctuates greatly, while the deep feature layer (conv4-6) can still locate the target accurately.

A siamese network structure based on multi-attention mechanism is used to track targets more robustly, the architecture is shown in Figure 2. In practical scenarios, accurately constructing the target representations is a difficult mission in UAV tracking. According to [18], features in the earlier layers of neural networks retain fine-grained details related to texture and shape, while deeper-level features can encode semantic abstractions that are invariant to nuisance variables. As shown in Figure 3, when there are appearance variations and disturbances from similar targets or backgrounds, the feature maps of UAV targets generated by conv2-3 vary significantly over time. In contrast, the object’s features in conv4-6 and conv5-3 are distinguished from the background region despite obviously being background changes. This property allows us to handle appearance variations and capture the object accurately. In summary, it is a challenge for a shallow network to counter UAVs with various sizes and variable presentations. A deeper network structure is necessary for precise localization and size estimation. Among popular deep neural network models such as ResNet, ResNeXt, and MobileNet, ResNet50 is chosen in this paper. We utilize an improved version of SiamRPN++ [18] as the baseline for UAV target tracking, which consists of a feature extraction network and a region proposal network (RPN). As shown in Figure 2. The AM in the structure is the added attention module, which will be described in detail in Section 3.3. The Siamese network has two main branches: the template branch and the search branch. The template branch extracts target features from the first frame of the input video as template z, while the search branch extracts image feature x from the search region of subsequent frames and measures its similarity with the template z using the cross-correlation function as shown in Equation (1).

(1) $$f\left(z,x\right)=f\left(\phi \left(x\right),\phi \left(z\right)\right)+b\cdot I$$

RPN is made up of a classification branch and a regression branch, which are responsible for classifying the foreground and background and regressing the target location, respectively. More details about RPN can be found in [10]. Target tracking demands the accurate classification of the object and distractors, but the appearance variations and motion blur may cause tracking to drift or even fail. Feature maps generated from different level layers of convolutional network contain multiple-scale information. This rich hierarchical information reserves semantic abstraction and fine-grained details, which is beneficial for robust tracking. According to SiamRPN++ [18], we aggregate multilevel convolution features from the last three residual blocks in ResNet50 to provide more discriminative representations, and these features are fed into three RPN modules separately, then the weighted sum is adopted directly on the RPN output, as shown in Equation (2).

(2) $$R_{out}=\overset{5}{\underset{l=3}{\sum }}\alpha_{l}\cdot R_{l}\;,\;C_{out}=\overset{5}{\underset{l=3}{\sum }}\beta_{l}\cdot C_{l}$$

### 3.3. Multiple Hybrid Attention Module

**Figure 4:**
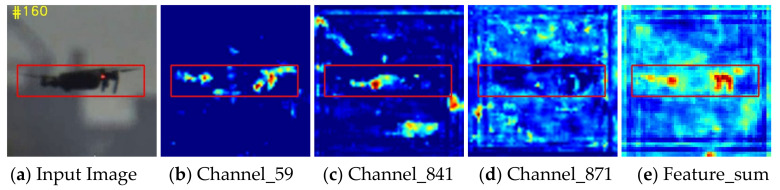
Visualization of feature maps of conv4-6. The first column: input images, the second to fourth columns: feature maps from different channels, the fifth column: the sum of the feature maps in equal proportions. It can be seen that not all features are useful for targeting; information on some channels (Channel-871) or positions (Channel-841) is sometimes redundant.

**Figure 5:**
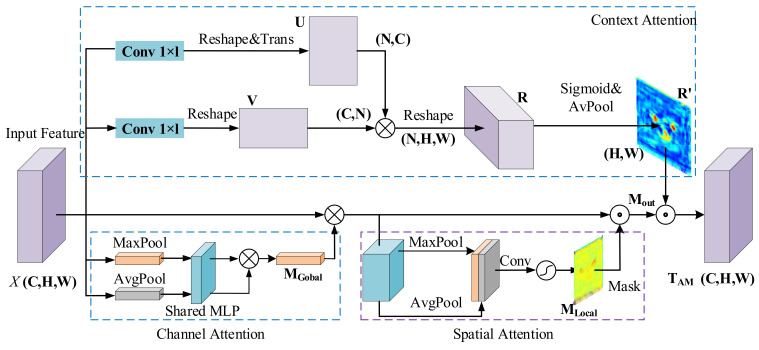
The proposed multi hybrid attention module consists of three submodules: channel attention, spatial attention, and context attention.

Anti-UAV videos have a wide field of view, the background is complicated, and there may be many disturbances that are similar to the target. The performance of the tracker is prone to be disturbed by the interference of meteorological factors, such as clouds or fog. In addition, not all of the multilevel features extracted from ResNet are helpful to boost the capability of the model, and some redundant information may lead to the decline of tracking properties, as shown in Figure 4. Thus, one multiple hybrid attention module is introduced to suppress background interferences and remove the negative impact of redundant features. The attention module is proposed to enhance the learned discriminative representations of ResNet50. As shown in Figure 5, the attention module consists of two parts: (1) channel and spatial attention module (CSAM), and (2) context attention module (CAM).

Specifically, CSAM includes both channel attention and spatial attention. Channel attention is utilized to adaptively reweight the features of each channel; it can decrease the weight of misleading channels while increasing the weight of reliable channels. On the other hand, spatial attention selectively enhances the informative differences between features at different spatial locations. Furthermore, CAM pays more attention to semantics between subregions of feature maps to provide more discriminative clues about the target.

(1) Channel and spatial attention module:

On the one hand, the features generated by a deep neural network contain a large amount of channel information. Different channel characteristics contribute differently to target expressiveness. Similarly, the feature maps also contain background information, and the complicated and changeable external information will interfere with the target location results. Consequently, the weights of feature maps on both spatial and channel dimensions are required to be distributed selectively. Inspired by [39], we introduce the channel and spatial attention module to suppress redundant information and highlight foreground features.

**Channel attention** aggregates channel information from the feature map *X* by employing average-pooling and max-pooling operations. In this way, we can obtain two one-dimensional global context descriptors: $u_{a}$ and $u_{b}$. Both descriptors are fed to a fully connected network with shared parameters to generate global channel attention weights. The attention mask is formulated as:

(3) $$u_{a}=Avgpool\left(F\right)\;,\;u_{b}=Maxpool\left(F\right)$$

(4) $$M_{Global}\left(X\right)=\sigma \left(F_{1}\left(F_{0}\left(u_{a}\right)\right)\right)+\sigma \left(F_{1}\left(F_{0}\left(u_{b}\right)\right)\right)$$

where $u_{a}$, $u_{b}\in \;{\mathbb{R}}^{C\times 1\times 1}$, and $M_{Clobal}\in \;{\mathbb{R}}^{C\times 1\times 1}$ denote the channel weights. The shared network is MLP with one hidden layer and $F_{0}\in \;{\mathbb{R}}^{C\times C}$ and $F_{1}\in \;{\mathbb{R}}^{C\times C}$ are the network parameters.

**Spatial attention** leverages average-pooling and max-pooling to compress the feature map along the channel dimension, generating two 2D feature maps: $f_{a}$ and $f_{m}$. Then, the feature maps are convoluted by a single-layer convolution network to generate a local spatial attention map $M_{Local}$:

(5) $$M_{Local}=\sigma \left(F_{3}\left(Maxpool\left(X⊗M_{Global}\right);Avgpool\left(X⊗M_{Global}\right)\right)\right)$$

(6) $$M_{out}=M_{Local}⊙\left(X⊗M_{Global}\right)$$

where σ denotes the sigmoid function, and the size of ML should be equal to feature map X in the spatial dimension.

(2) Context Attention Module:

Each specific target is usually related to its surroundings, and utilizing this contextual information can help to improve tracking accuracy. In the meantime, the correlation between each pixel in the image and its neighboring pixels also contains essential clues. Inspired by [40], in order to fully utilize the semantic information in the image, we proposed CAM based on the self-attention mechanism, as illustrated in Figure 5. CAM is capable of learning the correlation between different pixels. First, by feeding the feature vectors from the output layer $X\in \;{\mathbb{R}}^{C\times H\times W}$ into two parallel 1×1 convolutional layers, we obtain:

(7) $$U=W_{u}^{T}X\;,\;U=W_{v}^{T}X$$

where$\;U,V\in \;{\mathbb{R}}^{C\times H\times W}$ denote the two output feature vectors corresponding to the input signal X. Then, we reshape U and V to ${\mathbb{R}}^{C\times N}$, where N=H×W. To obtain the self-attention weights, we compute the relation matrix $M\in \;{\mathbb{R}}^{N\times N}$ by:

(8) $$M=U^{T}V$$

Note that when computing the similarity weights between local feature maps *U* and *V*, the self-attention module mainly utilizes the intrinsic correlation among the maps and learns their mutual dependence. We reshape M to $M\in \;{\mathbb{R}}^{N\times H\times W}$. Then, we utilize average pooling and sigmoid function to normalize the weights:

(9) $$M_{Context}=\sigma \left(Avgpool\left(M\right)\right)$$

where $M_{Context}\in \;{\mathbb{R}}^{H\times W}$. Configuration of the attention modules: note that there are three attention modules, including channel, space, and context. The channel–space sequence is put in parallel with the context module, so the output of the attention module is calculated by:

(10) $$T_{AM}=M_{Context}⊙M_{out}$$

where $M_{out}\in \;{\mathbb{R}}^{C\times H\times W}$ is the output feature of CSAM. In the end, we compute the dot product between the context attention matrix $M_{Context}$ and the feature maps $M_{out}$ to find the attention representation $T_{AM}\in \;{\mathbb{R}}^{C\times H\times W}$.

### 3.4. Reliability Discriminator

In the process of long-term tracking, the UAV target occasionally disappears and then shows up again. In addition, when the camera moves simultaneously with the target, rapid variations in illumination and environment can also affect the robustness of the algorithm. Therefore, it is necessary to accurately determine whether the target is lost so that the detection module can be restarted in time.

In this section, we first discuss how to utilize the response of RPN to estimate the reliability of tracking results. The classification branch of RPN is designed for foreground or background classification. If there are k anchors, the classification branch will generate 2k response maps, which indicate for negative and positive activation of each anchor, respectively, as illustrated in Figure 2 (there we set the number of anchors to 5). The anchor with the highest probability of belonging to the positive sample will be regarded as the target location. The probability score fluctuates within a certain range and is much lower when the tracking result is untrusted, and it is computed by:

(11) $$S_{p}=max\left(Softmax\left(z_{0},z_{1}\right)\right)$$

where $z_{0}\;,\;z_{1}\;$denotes the positive and negative activation for each anchor. We specify a threshold for each video. Concretely, the historical $S_{p}$ values are combined into an ensemble $\;C_{s}=\left\{S_{p}^{1},S_{p}^{2}\ldots S_{p}^{i}\right\}$ and the mean of the ensemble $C_{s}$ is represented by $M_{s}$. Defining a coefficient $\;O_{s}$ for $\;M_{s}$, we determine the tracking result unreliable when $S_{p}^{t}<O_{s}\cdot \;M_{s}\;$.

**Figure 6:**
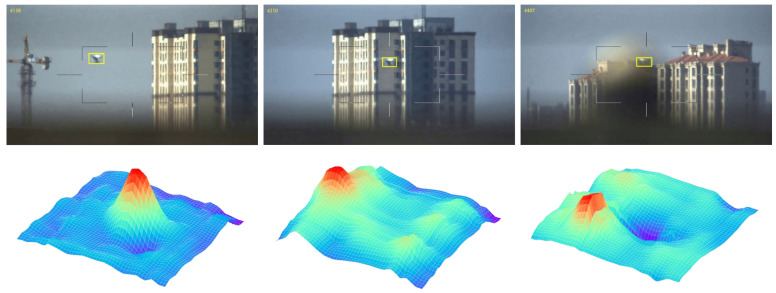
From top to bottom: input image and the response map of RPN. It can be seen that when the target is obscured or disturbed, the value of the peak will fluctuate dramatically.

In the correlation-filter-based methods, the peak-to-sidelobe ratio (APCE) is an indicator to quantify the sharpness of the correlation peak. As shown in Figure 6, if the APCE value is low, the correlation between the search and template may not be strong. We introduce APCE to SiamRPN++ [18], and it is computed by:

(12) $$\mathrm{APCE}=\frac{{\left|F_{\max}-F_{\min}\right|}^{2}}{mean\left(\sum_{w,h}{\left(F_{w,h}-F_{\min}\right)}^{2}\right)}$$

where $F_{\max}$ and $F_{\min}$ are the maximum response and the minimum response of the current frame, respectively. $F_{w,h}$ denotes the element value of the *w*th row and the *h*th column of the response matrix. The response map is usually only one channel in the correlation-filter- or SiamFC-based method. While the classification branch of the RPN module will produce response maps with 2k channels. Therefore, the APCE calculation method used for the correlation-filter-based tracker cannot directly apply here, so we compute a weighted sum of response maps produced by RPN.

(13) $$F=\overset{2k}{\underset{i=k+1}{\sum }}\hat{\alpha_{i}}M_{i}-\overset{k}{\underset{j=1}{\sum }}\hat{\beta_{j}}M_{j}$$

(14) $$\hat{\alpha_{i}}=\frac{\alpha_{i}}{\sum_{p=k+1}^{2k}\alpha_{p}},\;\;\hat{\beta_{j}}=\frac{\beta_{j}}{\sum_{q=1}^{k}\beta_{q}}$$

where $M_{i}$,$M_{j}$ denote the response maps of foreground and background activation, respectively. $\hat{\alpha_{i}}$ and $\hat{\beta_{j}}$ denote the adaptive weights for normalization, which are the maximum activation values of each channel. The reason behind this normalization procedure is that each response map reflects the shape similarity between its corresponding anchor and the actual target. As a higher activation value indicates higher similarity, this map should be allocated with a higher weight. Similar to the $S_{p}$ score, we compute the APCE scores and record them into a pool $\;C_{a}=\left\{S_{a}^{1},S_{a}^{2}\ldots S_{a}^{i}\right\}$; the $S_{a}^{i}$ denotes the *i*th frame’s APCE score and $M_{a}$ is defined as the average value of the pool $\;C_{a}$. The current APCE score $S_{a}^{t}$ is considered to be evidently low if $S_{a}^{t}<O_{a}\cdot \;M_{a}$. If that is the case, we believe that the tracking result is insecure, and then the redetection module should be activated.

In summary, we utilize both $S_{p}$ and APCE as indicators to evaluate the tracking state. The tracking result is only considered reliable when both their values exceed the specified threshold. For a particular tracking procedure, when unreliable results appear, the values for $S_{p}$ and APCE will be much lower than usual. The tracking result is only considered correct if the following reliability judgments are satisfied:

(15) $$\left\{\begin{array}{c} S_{s}^{t}>O_{s}\cdot M_{s}\\ S_{a}^{t}>O_{a}\cdot M_{a} \end{array}\right.$$

### 3.5. Redetection Module and Template Updating

(1) Redetection Network

Redetecting the target when tracking fails is a standard procedure in the long-term tracking framework, so it is essential to have a stable redetection module in our work. In this paper, we choose the well-known YOLOv5 as the redetection network. YOLO is a one-stage detection method. Its main idea is to divide the input image into a fixed number of cells and obtain region proposals by predicting the bounding box and the corresponding score for each cell. Finally, non-maximum suppression (NMS) is adopted to obtain the final detecting result. Compared with its earlier version, YOLOv5 has fewer parameters, faster speed, and higher accuracy, which makes it suitable for single-object detection.

**Figure 7:**
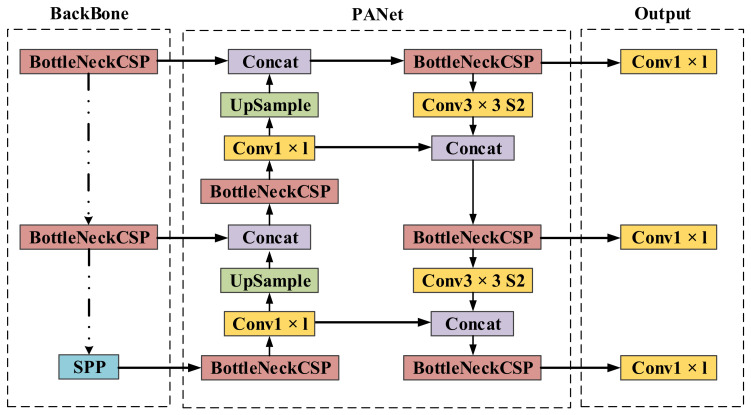
The architecture of YOLOv5, which consists of backbone and PANet.

As illustrated in Figure 7, the input of the network is an RGB image with dimensions of 3 × 608 × 608, and the output is a feature map with dimensions of 32 × 304 × 304. The network is mainly composed of CBL and CSPNet modules. CBL consists of two-dimensional convolution, batch normalization, and LeakyReLU activation function. CSPNet is composed of two-dimensional convolution, res unit and CBL module, and it is mainly responsible for enhancing the learning capability of CNN and ensuring accuracy while being lightweight.

(2) Template updating (Updating Discriminator)

The conventional updating method applied for template-based trackers is to update at each frame or with a fixed interval. However, this is computationally expensive and also prone to disturbance. Therefore, we exploit an updating discriminator for activating the updating module, and adopt an adaptive updating strategy based on historical information about UAVs.

First, the algorithm needs to know when to update. We design an updating discriminator and divide it into two different cases according to whether the tracking result is reliable or the detecting result is reliable. APCE is utilized to evaluate the quality of the tracking result. As APCE reflects the level of fluctuation of the response maps from RPN, a higher value indicates low noise and disturbance, so the tracking result is more likely to be reliable. When the APCE value exceeds the specified threshold, the template will be updated based on the current result. On the other hand, when the redetection module is activated, it indicates an obvious change of appearance, so the template must update to match the new aspect. YOLO network can output the score of the detection result. Consequently, we will also update the template when the YOLO score is larger than a certain threshold. Thus, we establish a startup mechanism for the updating module: UDC.

Then the model must generate the new template. Many prior works replace the template with the previous tracking result, which would cause the accumulation of noise, and finally lead to drifting. To address this problem, we adopt an adaptive-weight template computation model:

(16) $${\tilde{T}}_{i}=\lambda_{0}^{i}T_{0}^{GT}+\lambda_{1}^{i}{\tilde{T}}_{i-1}+\lambda_{2}^{i}T_{i}$$

where ${\tilde{T}}_{i}$ denotes the updated template at the current frame, $T_{0}^{GT}$ is the target template at the initial frame, ${\tilde{T}}_{i-1}$ is the template generated from history information, and $T_{i}$ denotes the template at the current frame.$\;\lambda_{0}^{i}$, $\lambda_{1}^{i}$, and $\lambda_{2}^{i}$ are adaptive weights which are determined based on the values of the APCE score and YOLO score. When the APCE value exceeds the prescribed value, we adjust the weight to:

(17) $$\lambda_{2}^{i}=\frac{\left(1-\lambda_{0}^{i}\right)\cdot S_{a}^{i}}{mean\left\{S_{a}^{i-1}\right\}+S_{a}^{i}},\lambda_{1}^{i}=\left(1-\lambda_{0}^{i}\right)\cdot \left(1-\lambda_{2}^{i}\right)$$

where $S_{a}^{i}$ is the APCE value at the current frame, and the set $\left\{S_{a}^{i-1}\right\}$ contains the confidence level information for all historical templates. The activation of the redetection module indicates radical changes in the appearance compared to the historical template, so we reduce the weight of ${\tilde{T}}_{i-1}$:

(18) $$\lambda_{2}^{i}=\left(1-\lambda_{0}^{i}\right)S_{\mathrm{YOLO}},\;\lambda_{2}^{i}=\left(1-\lambda_{0}^{i}\right)\cdot \left(1-S_{\mathrm{YOLO}}\right)$$

where $S_{\mathrm{YOLO}}$ is the detection score of YOLO.

## 4. Experiments

### 4.1. Benchmark

As a high-quality benchmark in dynamic environments, an anti-UAV [41] dataset was proposed at the 1st International Workshop on Anti-UAV Challenge at CVPR 2020. It consisted of over 300 video pairs (both RGB and infrared) and annotated more than 580k bounding boxes, spanning multiple occurrences of multiscale UAVs (i.e., large, small and tiny), mainly from DJI and Parrot to guarantee the diversity of data. In total, 318 video pairs, 160 are divided into the training set, 91 are assigned to the test set, and the rest are applied as the validation set. The videos recorded include two lighting conditions (day and night), two light modes (infrared and visible), and diverse backgrounds (buildings, clouds, trees, etc.); there are more video sequences captured in the day. Each video is stored in an MP4 file with a frame rate of 25 FPS. To be specific, Anti-UAV sets a series of challenges, including out-of-view, occlusion, fast motion, scale variation, low illumination, thermal crossover, and low resolution. Anti-UAV is the first constructed anti-UAV benchmark for capturing real dynamic scenes in the community. More and more work begins to be performed on the challenging dataset.

### 4.2. Implementation Details

In the training stage, The SiamRPN [9] and YOLOv5 networks are trained offline. When training SiamRPN [9], a three-stage learning strategy is adopted. The Siamese network is pre-trained on the sets of YouTube-BoundingBoxes [42], ImageNet VID, ImageNet [43], and COCO [44] datasets firstly. Then the parameters of the backbone are fixed, and a hybrid attention module is introduced into the base structure. Next, the hybrid attention module and RPN are retrained through the Anti-UAV [41] training subset. Finally, the whole network is trained end-to-end on Anti-UAV to optimize parameters. In YOLOv5’s training stage, we just adopt the anti-UAV training set since the detection algorithm only needs to distinguish foreground and background. The network is trained by the stochastic gradient descent method (SGD). The proposed algorithm is evaluated on anti-UAV testing subset, which contains 91 RGB videos. Our algorithm is compared with 9 recent SOTA trackers: MDNet [6], SiamDW [12], SiamFC [7], SiamRPN [9], SiamRPN++ [18], TransT [45], SiamCAR [46], SiamRPN++LT [18] and CLNet [47]. Two metrics were introduced to evaluate the performance of all methods: distance precision (DP) at the threshold of 20 pixels, the area under the curve (AUC) of success plot. And scores in the precision plots were defined as the percentage of the frames in which the center position errors are smaller than the predefined threshold. Meanwhile, scores in the success plots indicate the percentage of the frames in which the overlap rate of the tracking area and the boundary frame is larger than the threshold. In both training and testing phases, we employ equal-sized patches with 127 pixels for template and patches with 255 pixels for the search areas. As for reliability discriminator, $O_{s}$ is set to 0.8 and $O_{a}$ is set to 0.35. The proposed framework is implemented in Pytorch, and experiments were performed on NVIDIA GTX 3090 GPU.

### 4.3. Comparison with SOTA Trackers

**Figure 8:**
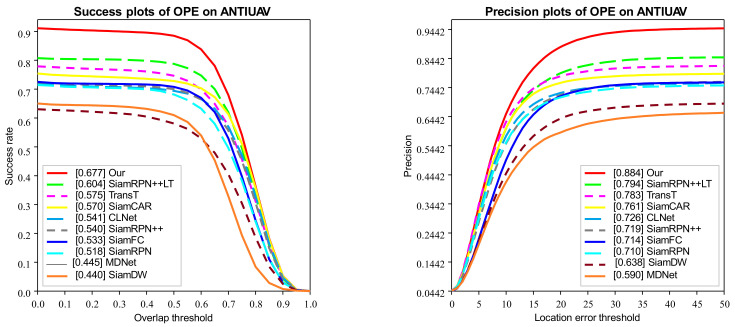
Success and precision plots of OPE on Anti-UAV. Plots show the comparisons of our algorithm with other SOTA trackers.

We utilize One-Pass-Evaluation (OPE) to evaluate the effectiveness of our anti-UAV long-term tracking framework, and the comparison with other SOTA trackers is performed on Anti-UAV benchmarks. The results of success plots and distance precision plots are illustrated in Figure 8. And their distance precision (DP) at the threshold of 20 pixels, overlap precision (OP) at the threshold of 0.5 are shown in Table 1. Among these trackers, our SiamAD achieves the best result in AUC (67.7%) and DP (88.4%). Compared with the second place SiamRPN++LT [18], SiamAD achieves an improvement of 7.3% and 9.0% in AUC and DP, respectively. Furthermore, compared to the baseline SiamRPN++ [18], our tracker also achieves significant improvements, with a relative gain of 13.7% on success rate and a relative gain of 16.5% on accuracy rate. Notably, SiamAD reaches a speed of 38.8 FPS to meet real-time tracking requirements. These results demonstrate that the proposed multiscale feature extraction network, hybrid attention modules, and the hierarchical discriminator can work together to achieve an efficient and accurate UAV-object-tracking capability.

Meanwhile, we can find that the long-term trackers are more likely to gain higher competence. SiamRPN++LT [18] shows excellent performance with an OP of 78.8% and a precision rate of 79.4%, which are significantly higher than the short-time tracker. In conclusion, we believe that trackers based on long-term tracking or detection are more competitive for UAV-tracking tasks.

### 4.4. Attribute-Based Evaluation

To analyze the tracking performance under different challenge factors comprehensively, the attributed-based evaluation is conducted. Anti-UAV [40] provides attribute annotations for each video to help identify the pros and cons of trackers in dealing with various difficulties. These annotations include out-of-view (OV), occlusion (OC), fast motion (FM), scale variation (SV), low illumination (LI), thermal crossover (TC) and low resolution (LR). In the test set, OV and FM appear frequently and are also difficult missions during UAV tracking.

**Figure 9:**
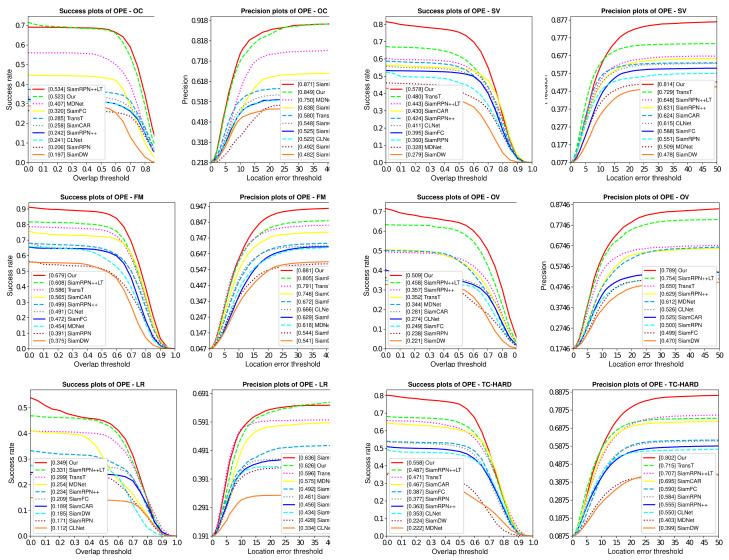
Attribute-based evaluation on Anti-UAV benchmark. The success and precision plots are shown six challenging factors.

Figure 9 illustrates the tracking success rate and accuracy rate under different challenge factors. As we can observe, SiamAD performs much better than other trackers in OV, SV, and FM. In the case of out-of-view, our proposed method outperforms SiamRPN++LT [18] with an improvement of 5.2% in AUC and 4.0% in DP. In the case of scale variation, SiamAD achieves a relative gain of 8.2% in AUC and 7.2% in DP compared to TransT [45]. The reasons for the significant performance improvement can be summarized in two aspects: on the one hand, the single category redetection module based on YOLO has a high detection capability for the identified targets and can accurately re-locate them after they have been lost; on the other hand, the updating method of the target template based on UDC can significantly improve the accuracy of the tracking network. SiamAD also offers outstanding performance in OC and LR. Due to the hybrid attention module, the model’s ability to express features and resist interference is strengthened. As a result, the tracker can handle difficulties caused by complex environments and the changes of UAV appearance more efficiently.

The number of times in which the re-detection is triggered is obtained and recorded in Table 2. During the test, the redetection module was activated at a rate of 9.2%. In general, the probability of the redetection being activated increases when dealing with challenge factors, especially when the UAV is difficult to observe (LR, OV, and OC). This illustrates the necessity of detection in response to the missing object.

### 4.5. Qualitative Analysis

**Figure 10:**
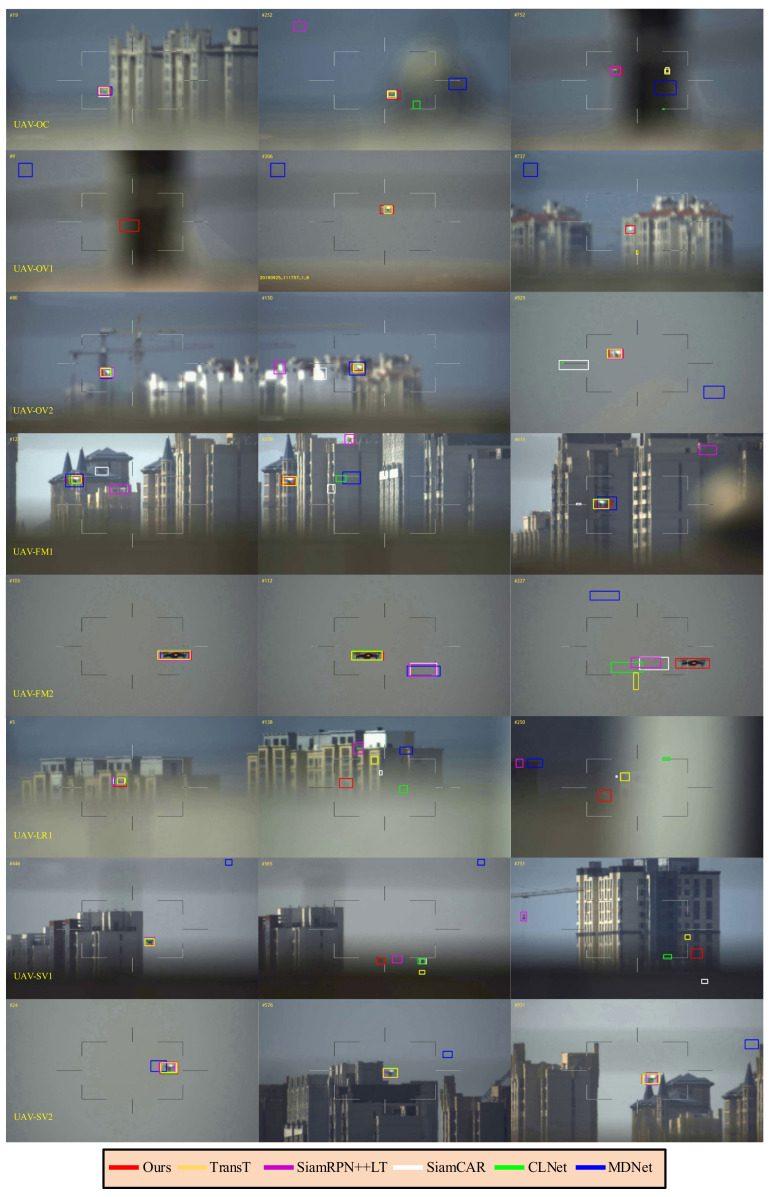
Qualitative evaluation of SiamAD, MDNet [5], CLNet [47], SiamCAR [46], SiamRPN++LT [18], and TransT [45] on eight challenging sequences. We name these videos, from top to bottom: UAV-OC, UAV-OV1, UAV-OV2, UAV-FM1, UAV-FM2, UAV-LR, UAV-SV1, and UAV-SV2, respectively (Reprinted with permission from Ref. [41]. 2022, Jiang et al.).

The proposed method is qualitatively compared with five state-of-the-art trackers MDNet [5], SiamRPN++LT [18], SiamCAR [46], TransT [45], and CLNet [47]. Figure 10 shows the results of eight challenging videos which from the Anti-UAV benchmark.

**Occlusion (OC)**. Due to the complex and changeable environment and the fact that UAVs are generally small, UAV objects are prone to being blocked by buildings or vegetation for long periods. In the UAV-OC sequence, the object is completely occluded in frames 224–262. When the UAV reappears, TransT [45] and SiamAD locate it first. Although CLNet [47] can capture the target as well, the positioning accuracy is significantly lower than other methods because the model does not update. SiamRPN++LT [18] is a long-term tracker and depends on the robustness of the pre-trained network to redetect the object. After the target is lost, the tracker can quickly relock it. After the second occlusion at frame 753, only SiamAD and SiamRPN++LT [18] are able to capture the target again.

**Out-of-View and Fast Motion (OV & FM)**. OV is one of the most challenging factors in UAV tracking tasks, and methods demand prompt detection of the object when it reappears. OV is present in the UAV-OV1, UAV-OV2, and UAV-FM2 sequences. At the beginning of UAV-OV1, the target is just out of view. This means that trackers do not have access to the position and appearance of the target in the initial frame. Therefore, almost all methods based on generative models (SiamCAR [46], TransT [45] and CLNet [47]) fail to track. However, relying on the template update mechanism and redetection module, SiamAD can gradually find the target. In the UAV-OV2 and UAV-FM2 sequences, the target leaves view and reappears at the end. This is a typical case during tracking UAVs. Similarly, only SiamRPN++LT [18] and SiamAD can re-locate the UAV.

In order to capture the object when it accelerates, a larger search area should be applied. However, this may increase the risk of introducing jamming signals. In the UAV-OV2 and UAV-FM2 sequences, the movement of the UAV and the rotation of the camera are superimposed over one another, and such a complex motion causes the target to shift a significant distance of more than 60 pixels between two adjacent frames. The target overlaps with the background and changes suddenly. In UAV-FM1, SiamRPN++ [18] and SiamCAR [46] lose their targets first due to interference from similar backgrounds. Although MDNet adopts the online update strategy to adjust the network parameters, the introduction of erroneous features still resulted in deviations in the localization. Combined with the hybrid attention mechanism and feature fusion methods, SiamAD and TransT are effective in suppressing redundant information and enhancing the discriminative ability of the feature representations. This allows the trackers to resist the effects of similar interference in the rapidly changing background and effectively improves tracking accuracy.

**Low Resolution (LR)**. UAV-LR demonstrates UAV tracking results in low visibility conditions. The main challenge is low resolution and low signal-to-noise ratio in this video. MDNet [7] is less effective due to the introduction of noise during online updates. In some simple scenes, the influence of low resolution on tracking results is easy to overcome. Nevertheless, when combined with background clutter, the tracking bounding box can easily drift to similar disturbances in the vicinity of the target. It is difficult for trackers such as TransT [45] and SiamCAR [46] to obtain accurate target representations in such conditions, which makes the tracking results deviate from the background. For instance, SiamCAR [46] could intermittently re-locate to the UAV but quickly lost the target again since the appearance variations. SiamAD leverages the re-detection mechanism to solve the problem of the drift of the bounding box. Simultaneously, introducing a template update strategy with high confidence can effectively avoid the templates being contaminated.

**Scale Variation (SV)**. When there are no other challenge properties, the algorithms tested in this paper are all well adapted to UAV scale changes. Algorithms such as SiamCAR [46] and SiamRPN++ [18], which are based on RPN, all exhibit favorable adaptive perceptions of target scale estimation.

As discussed above, the proposed SiamAD performs well in the above videos. Its main achievement can be summarized from the following three aspects:

(1) While most trackers are prone to failure when dealing with challenges such as severe occlusion or out-of-view; SiamAD still achieves good tracking results which benefit from the accurate judgment on the reliability of tracking and reliable redetection results from the YOLO network.

(2) Our SiamAD is effective in handling distractors of background and appearance variations. This is because we introduce attention mechanisms and multiscale feature fusion based on the deep Siamese network. The feature maps extracted from the network can represent rich semantic information and enhance the discriminability of the model.

(3) The redetection module provides high-quality templates for algorithm updating, and SiamAD utilizes all reliable features to generate a robust template with rich information. Therefore, our proposed method can adjust adaptively to the variations of environment and improve the tracking performance in complex scenes.

### 4.6. Ablation Studies

To quantitatively evaluate the effectiveness of each component of our SiamAD, we conduct several experiments on Anti-UAV with various components, including the multi-hybrid attention module proposed in Section 3.3, the hierarchical discriminator proposed in Section 3.4, and the redetection module and updating strategy proposed in Section 3.5. As illustrated in Table 3, the tracker being equipped with the attention mechanism or redetection significantly strengthens the performance compared to the original base tracker. The introduction of updating online has also contributed to improving the model. In the following paragraphs, we describe the impact of each part in detail.

**Hybrid attention module**. As mentioned in Section 3.3, we introduce two attention modules to the Siamese network, namely CSAM and CAM. Based on SiamRPN++ [18], we introduce our attention modules gradually and validate them on anti-UAV [41] test set. As shown in Table 4, the model contained with both CSAM and CAM achieved the best tracking performance. Furthermore, we found that the introduction of CAM showed a significant improvement in tracking success rate and precision rate, increasing by 3.0% and 4.8%, respectively. This illustrates that the association information between the foreground and background of the object is helpful to enhance the representation of features. CSAM also achieved 2.5% and 2.6% improvement in success rate and accuracy, respectively, which also improved the tracking performance.

**Figure 11:**
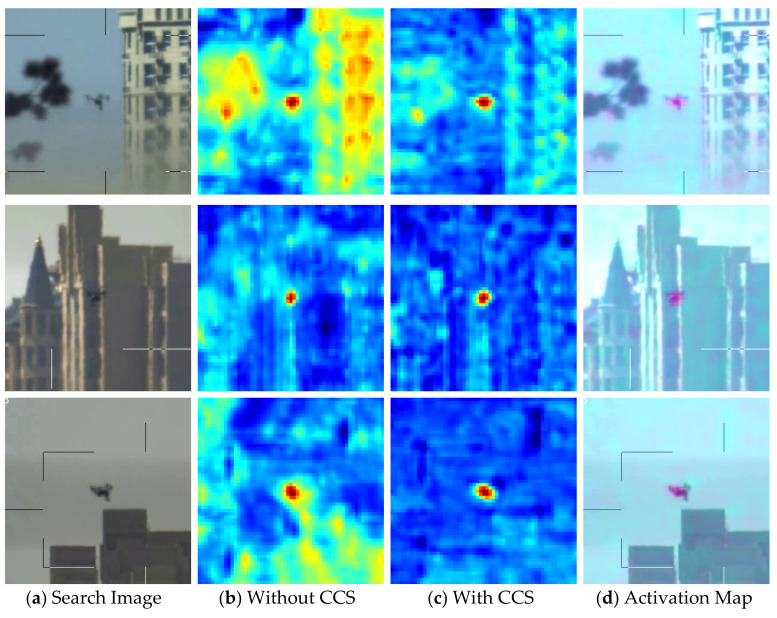
Visualization of response maps. The first column: search images; the second column: response maps without our MHAM Module; the third column: response maps with MHAM Module; and the fourth column: attention activation map which illustrates the region of interest.

As shown in Figure 11, with our MHAM module, the response maps of networks focus on UAV object more accurately. MHAM can effectively screen target features, enhancing the network’s ability to discriminate between object and distractors.

**Reliability discriminator for re-detection.** To justify the impact of the redetection mechanism in our framework, we conduct an ablation study where we compare tracking results with and without redetection networks, and experimental data are presented in Table 3. Note that the experiments are conducted using Siamese networks incorporated with attention modules. The results indicate a 7.8% increase in AUC and a 9.8% increase in DP when incorporated with a redetection network—a significant improvement in tracking effectiveness. In addition, we verify the impact of parameter settings of the reliability discriminator.$\;S_{p}$ (Equation (11)) and APCE (Equation (12)) are exploited as the evaluation indicators of the confidence of tracking results, then we select different combinations of coefficients $O_{s}$ and $O_{a}$ (Equation (15)), as shown in Table 5. We find that large values of those coefficients are beneficial to tracking accuracy, but in practice, the real-time performance will become worse. Furthermore, the performance of this model is not optimal when we set same thresholds for all videos. We interpret that adaptively adjusting threshold according to the properties of video is more conducive to fully exploiting the redetection module’s capabilities.

**Figure 12:**
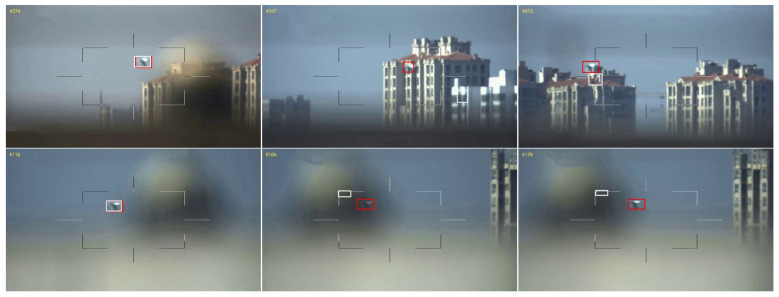
The tracking results of the redetection module on the Anti-UAV benchmark. The red bounding box represents the tracking results of SiamAD configured with a redetection module, while the white bounding box indicates the results of the tracker without redetection.

To further demonstrate how re-detection module works on our tracker, we select two typical videos from Anti-UAV for the comparison experiment, SiamRPN++ [18], and our SiamAD. We name these two videos *UAV-VIDEO1* and *UAV-VIDEO2*, respectively, and the results are shown in Figure 12.

(1) In *UAV-VIDEO1*, the UAV object is disturbed by buildings in the background. SiamRPN++ [18] fails to track the target since the color of the background and target is difficult to differentiate. However, our redetection module is unaffected by interference sources and is able to correctly locate the target in this video.

(2) In *UAV-VIDEO2*, the UAV target is seriously occluded; both trackers lose target at the same time. When the object reappears, SiamRPN++ [18] is incapable of capturing the target immediately, but the redetection module can quickly identify and relock the UAV.

**Updating discriminator for template updating. Table 6** shows the performance of different template update strategies to illustrate how they affect the outputs. We compared three settings: no update for the template, generating a new template directly from redetection and using the template update method mentioned in Section 3.4. As can be seen in Table 3, template updates play an important role in improving tracking accuracy. Moreover, compared with the direct application of redetection results, the proposed template update strategy preserves the historical information of the object, and the generated template is more robust.

### 4.7. Testing and Analysis on UAV Tracking Video

**Figure 13:**
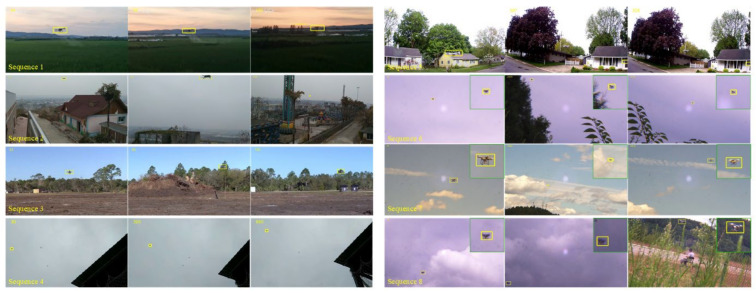
The tracking results of the SiamAD applied in real scenarios.

To quantitatively evaluate the effectiveness of SiamAD in real UAV tracking scenarios, more performance tests of the algorithm based on actual UVA videos are carried out. In order to ensure the accuracy of the experiments, videos of various specifications of UAVs in different application scenarios are collected. The majority of the test data is live videos with a resolution of 1920 × 1280 and a frame rate of 30 fps, and the shooting distance is approximately 200 mm. The tracking capability of our proposed algorithm can still be demonstrated in an actual test environment, and some tracking results are shown in Figure 13.

*Sequence 3* and *Sequence 5* show a UAV in a changing background. The target is moving rapidly, the image features are blurred, and there is background interference. The test results demonstrate that SiamAD is able to detect targets in complex and changing backgrounds and is also resistant to disturbances in practical applications. In Sequence 5, the high-speed movement of the UAV causes the tracker to lose the target frequently. However, the global search of the redetection module can make up for the failure of the tracking algorithm. Our SiamAD can handle object scale variations well (*Sequence 2*, *Sequence 7*) and also enables the tracking of small targets (*Sequence 4*), which benefits from the accurate estimation of bounding boxes by RPN. During testing, there are situations where the UAV undergoes heavy occlusion or leaves view (*Sequence 7*, *Sequence 8*), but SiamAD could accurately identify the entire target after the object reappeared.

**Figure 14:**
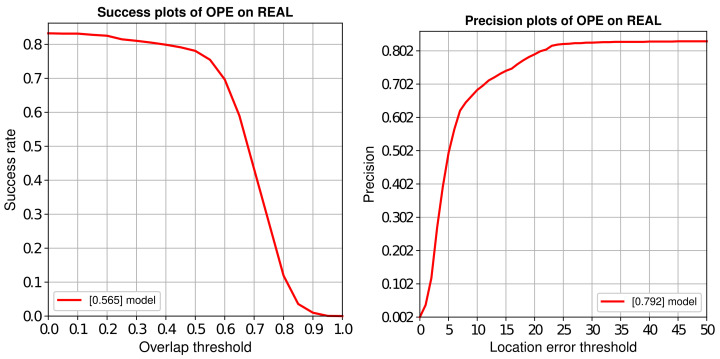
Success and precision plots of OPE on the homemade test set.

Some of the image samples were annotated and a small homemade test set was created. The OPE method is used to evaluate the accuracy of SiamAD. The results of success plots and distance precision plots are illustrated in Figure 14. On this test set, our SiamAD achieves an AUC score of 56.5% and a DP score of 79.2%. This proves that the method can still maintain a good success rate and accuracy when dealing with more challenging tracking conditions. Compared to the test results in Section 4.3, the performance of the algorithm on the homemade test set is reduced. This is mainly due to two factors. First, compared to Anti-UAV, the scenes of our video are more diverse, and the movement and appearance of the UAV vary more drastically. This makes the challenge for the proposed algorithm more difficult. Moreover, the redetection module is trained on a fixed dataset; the distributions of the self-made test set video and the Anti-UAV training set are very different. As a result, the performance of the detection algorithm based on the global search strategy is caused to degrade.

## 5. Conclusions

In this paper, a long-term tracking framework for anti-UAV is proposed. Our approach is developed based on both Siamese tracker and redetection networks and is equipped with two enhanced components: a hybrid attention module that promotes the attention distribution fitting degree by three series-parallel attention subnets and a hierarchical discriminator that adopts a flexible double threshold strategy on redetecting and precisely updating the current template. Additionally, a template updating strategy is exploited to further improve the tracking accuracy. The proposed work outperforms many state-of-the-art trackers on the Anti-UAV benchmark. We have demonstrated the effectiveness of our proposed modules, and the experimental results qualitatively demonstrate that each component is beneficial to performance improvement.

The proposed anti-UAV tracking method generally consists of a tracker, discriminator, redetection module, and updating module so that the tracker can maintain excellent performance in various challenging situations. Moreover, this algorithm also has a remarkable ability to track UAVs in actual scenarios. In the future, the existing framework can be optimized as follows. First, the search strategy of the redetection module can be refined and the detection and the tracking network could be fused to achieve network parameter sharing, making our method more efficient. Moreover, as the motion information of UAVs is not considered in this paper; a dynamic model based on spatiotemporal correlation can be established to improve the robustness of the tracking method. Lastly, the current research in this article is rarely concerned with LSS-target (Low Altitude, Slow Speed, Small Target). From a practical point of view, it is necessary to optimize the network for the problem of tracking low-altitude small targets. We hope that our work will encourage more advanced work in anti-UAV technology.

## Figures and Tables

**Table 1:** A performance comparison test of our approach with other SOTA trackers on Anti-UAV benchmark. Our SiamAD achieves the highest success and precision rate. The top three values are highlighted by red, green and blue.

Tracker	OP (%)	DP (%)	Where	When
SiamDW	61.0	63.8	CVPR	2019
MDNet	58.1	59.0	CVPR	2016
SiamRPN	69.2	68.0	CVPR	2018
SiamFC	71.0	71.4	ECCV	2016
SiamRPN++	70.2	71.9	CVPR	2019
CLNet	69.5	72.6	ICCV	2021
SiamCAR	72.4	76.1	CVPR	2020
TransT	74.5	78.3	CVPR	2021
SiamRPN++LT	78.8	79.4	CVPR	2019
SiamAD (Ours)	88.2	88.4		

**Table 2:** The number of times in which the redetection is triggered.

Factors	OV	OC	FM	SV	LR	ALL
Redetecton_ratio (%)	31.7	30.4	12.8	22.9	46.0	9.2

**Table 3:** Ablation study of our approach on the Anti-UAV benchmark in terms of success and precision. The highest values is highlighted by bold.

Tracker	AUC (%)	DP (%)
Baseline tracker (SiamRPN++)	54.0	71.9
Only with attention modules	59.9	78.6
Only with re-detection	60.9	79.7
Attention modules + re-detection	65.5	85.7
With adaptive update	**88.2**	**88.4**

**Table 4:** A performance comparison test of our approach with different attention module on the Anti-UAV benchmark. The best values is highlighted by bold.

Baseline	+CSAM	+CAM	AUC (%)	DP (%)
SiamRPN++			54.0	71.9
	✓		56.5	74.5
		✓	57.3	75.4
	✓	✓	**59.9**	**78.6**

**Table 5:** The evaluation of the performance of redetection. Different thresholds are used to verify the impact. The highest values is highlighted by bold.

Tracker	AUC (%)	DP (%)	Speed (fps)
Fixed threshold	67.2	87.9	34.5
$O_{s}=0.9\;,\;O_{a}=0.4\;$	67.6	88.2	33.1
$O_{s}=0.8\;,\;O_{a}=0.35\;$	**67.7**	**88.4**	36.5
$O_{s}=0.7\;,\;O_{a}=0.30\;$	67.6	**88.4**	**37.1**

**Table 6:** The evaluation of the performance of different template update strategies. The highest values is highlighted by bold.

Tracker	AUC (%)	DP (%)
Without updating	65.5	85.7
Update by re-detection	66.9	87.7
Update adaptively	**67.7**	**88.4**

## Data Availability

The data presented in this study are not publicly available at this time but can be obtained from the authors.
